# Sex Pheromones and Reproductive Isolation in Five Mirid Species

**DOI:** 10.1371/journal.pone.0127051

**Published:** 2015-05-14

**Authors:** Chang Yeol Yang, Se-Jin Kim, Junheon Kim, Taek-Jun Kang, Seung-Joon Ahn

**Affiliations:** 1 National Institute of Horticultural and Herbal Science, Rural Development Administration, Wanju, Republic of Korea; 2 Institute of Agriculture and Life Science (BK21^+^), Gyeongsang National University, Jinju, Republic of Korea; 3 Department of Entomology, Max Planck Institute for Chemical Ecology, Jena, Germany; USDA-ARS, UNITED STATES

## Abstract

Mate location in many mirid bugs (Heteroptera: Miridae) is mediated by female-released sex pheromones. To elucidate the potential role of the pheromones in prezygotic reproductive isolation between sympatric species, we investigated differences in the pheromone systems of five mirid species, *Apolygus lucorum*, *Apolygus spinolae*, *Orthops campestris*, *Stenotus rubrovittatus* and *Taylorilygus apicalis*. GC/MS analyses of metathoracic scent gland extracts of virgin females showed that all five species produced mixtures of hexyl butyrate, (*E*)-2-hexenyl butyrate and (*E*)-4-oxo-2-hexenal, but in quite different ratios. (*E*)-2-hexenyl butyrate was the major component of *A*. *spinolae*, while hexyl butyrate was the most abundant component in the pheromone blends of the other four species. In addition to the three compounds, a fourth component, (*E*)-2-octenyl butyrate, was present in the gland extracts of *A*. *lucorum* and *T*. *apicalis* females. Field tests suggest that the ternary blends of hexyl butyrate, (*E*)-2-hexenyl butyrate and (*E*)-4-oxo-2-hexenal as found in the extracts of the females of each species do not inhibit attraction of conspecific males but ensure species-specificity of attraction between *A*. *lucorum*, *O*. *campestris* and *T*. *apicalis*. Furthermore, (*E*)-2-octenyl butyrate was essential for attraction of *A*. *lucorum* and *T*. *apicalis* males, but strongly inhibited attraction of male *A*. *spinolae*, *O*. *campestris* and *S*. *rubrovittatus*. The combined results from this study and previous studies suggest that the minor component and pheromone dose in addition to the relative ratio of the major components play an important role in reproductive isolation between mirid species.

## Introduction

Miridae are one of the most species-rich families of insects, with over 11,000 described species [1]. Females of many mirid species have been shown to attract males by means of long-range sex pheromones [2,3]. In Miridae, sex pheromones to date have been identified from 16 species [4]. These data show that mirid bugs generally use saturated and unsaturated short-chain esters and an unsaturated ketoaldehyde for mate finding. Of these, ten species utilize mixtures of hexyl butyrate, (*E*)-2-hexenyl butyrate and (*E*)-4-oxo-2-hexenal in their sex pheromones. In moths, species specificity of sex pheromone blends is responsible for pre-mating reproductive isolation between sympatric species [5–7]. However, there have been few studies on pheromonal mechanisms in maintaining reproductive isolation of sympatric mirid species.

Several mirid species are economically important pests of various agricultural crops in northeast Asia [8–11]. Identifying the sex pheromone of these species would lead to a useful monitoring tool and to an environmentally safe control by mating disruption, mass trapping or attract-and-kill tactics. In Japan, Yasuda et al. [12] reported that the female sex pheromone of *Stenotus rubrovittatus* consisted of hexyl butyrate, (*E*)-2-hexenyl butyrate and (*E*)-4-oxo-2-hexenal in a ratio of approximately 100:46:5. Recently, we identified these three compounds in a ratio of 20:100:7 from extracts of metathoracic scent glands of female *Apolygus spinolae*, and found that (*E*)-2-hexenyl butyrate and (*E*)-4-oxo-2-hexenal are essential for male attraction [13]. Similarly, a previous study conducted in China showed that *Apolygus lucorum* is attracted to a mixture of (*E*)-2-hexenyl butyrate and (*E*)-4-oxo-2-hexenal [14]. However, the binary blend was not attractive to male *A*. *lucorum* in Korea (Yang CY, personal observation).

Therefore, we decided to examine the sex pheromone system of the Korean population of *A*. *lucorum* by chemical analyses of metathoracic scent glands of females and field testing of sex pheromone components. While conducting field trials of attraction of male *A*. *lucorum* and *A*. *spinolae*, we noticed that males of three other mirid species, *Orthops campestris*, *S*. *rubrovittatus* and *Taylorilygus apicalis*, were captured in traps baited with some of the blends. We also examined the pheromonal system of the three mirids to determine the role of the pheromones as prezygotic reproductive isolating mechanisms between sympatric species.

## Materials and Methods

### Insect Material

Field tests and some bugs collections were carried out on private land and we confirm that the owner of the land gave permission to conduct the study on this site.

Nymphs of *A*. *lucorum*, *A*. *spinolae* and *T*. *apicalis* were collected from mugwort (*Artemisia princeps* Pampanini) growing in the vicinity of vineyards in Suwon, Korea (37.2°N, 127.1°E). In addition, nymphs of *O*. *campestris* and *S*. *rubrovittatus* were collected from Korean angelica (*Angelica gigas* Nakai) and rice (*Oryza sativa* Linne), respectively. The bugs were individually reared on their host plants in plastic bottles (7 cm high and 2.5 cm diameter), and maintained at 25°C under a L14:D10 photoperiod. After eclosion, bugs were sexed based on the presence or absence of an ovipositor groove on the ventral side of the abdomen and provided with a cotton pad soaked with a 10% sucrose solution.

### Chemicals

Synthetic hexyl butyrate was obtained from Pherobank (Wageningen, The Netherlands; >99% purity). (*E*)-2-Hexenyl butyrate (98.6% purity), (*Z*)-2-hexenyl butyrate (95.0% purity), (*E*)-2-octenyl butyrate (98.0% purity) and (*Z*)-2-octenyl butyrate (97.0% purity) were prepared by reaction of butyric anhydride with (*E*)-2-hexenol (Sigma-Aldrich, St. Louis, MO, USA), (*Z*)-2-hexenol (TCI, Tokyo, Japan), (*E*)-2-octenol (TCI, Tokyo, Japan) and (*Z*)-2-octenol obtained by hydrogenation of 2-octyn-1-ol using Lindlar’s catalyst, respectively, in the presence of pyridine. (*E*)-4-Oxo-2-hexenal (97.4% purity; 17.5% yield) was synthesized by treating 2-ethylfuran (Alfa Aesar, Heysham, UK) with N-bromosuccinimide and pyridine in aqueous tetrahydrofuran as described by Moreira and Millar [15]. *n*-Alkanes (C_14_-C_17_), dimethyldisulfide (DMDS) and dodecyl acetate (12:OAc) were purchased from Sigma-Aldrich.

### Extraction of Metathoracic Scent Glands (MSG)

Two to four day-old unmated female adults of each species were anesthetized with CO_2_ between hours 4 and 8 of the photophase. Thoraxes containing the MSG were dissected under the microscope with microscissors and forceps, and placed individually in a 0.3-ml conical glass vial (Wheaton, Millville, NJ, USA) filled with 10 μl of hexane. The supernatant of MSG extracts was transferred to another vial after 1 min due to rapid degradation of (*E*)-4-oxo-2-hexenal in solvent with macerated insect material [16] and stored in a freezer (–20°C) until GC/MS analysis.

### Chemical Analyses

GC/MS analyses of MSG extracts were carried out with an Agilent 6890 N GC interfaced to an Agilent 5975C mass-selective detector (Agilent, Santa Clara, CA, USA). Extracts were run on DB-Waxetr and DB-23 columns (30 m×0.25 mm×0.25 μm film thickness; J&W Scientific, Folsom, CA, USA). Injection was splitless and helium was the carrier gas (1 ml/min). Injector temperature was 250°C, and the column temperature was 40°C for 2 min, rising to 220°C at 10°C/min and then held for 10 min. Electron impact mass spectra were monitored at 70 eV in the mass range of 40–300 amu. Chemicals from the extracts were identified by comparison of their retention indices (RIs) relative to *n*-alkanes and mass spectra with those of authentic standards on the two columns. Quantities of compounds were estimated by using synthetic hexyl butyrate as an external standard.

Determination of double bond positions in unsaturated compounds of MSG extract was accomplished by reaction with DMDS. For the DMDS derivatization of a 10-female gland extract of *A*. *lucorum*, we followed the procedure described by Buser et al. [17]. The DMDS adduct was then analyzed by GC/MS on the DB-23 column under the same conditions as above.

### Field Trials

Field trials were carried out to compare attractiveness of the candidate pheromone components to males of *A*. *lucorum* and *T*. *apicalis* during May and June of 2014 in vineyards in Suwon, Korea, where many weeds including mugwort were growing between the grapes. Delta traps with sticky floors (28×20 cm; Green Agro Tech, Korea) were hung from branches of grape vines approximately 1 m from the ground. Traps were baited with high-density polyethylene tubes (OD 3 mm, ID 2 mm, length 10 cm; Shin-Etsu Chemical, Tokyo, Japan) loaded with candidate pheromones. It is known that the blend of hexyl butyrate, (*E*)-2-hexenyl butyrate, and (*E*)-4-oxo-2-hexenal emitted from the tubes is similar to the blend loaded [18]. Test compounds were dissolved in 12:OAc as solvent to give a total amount of 100 mg per tube and the antioxidant butylated hydroxytoluene was added at 1% to all solutions. Each tube was filled with test solutions and heat-sealed at both ends.

To examine attraction of male *O*. *campestris* to candidate pheromones, two field trials were conducted during June and July of 2014 in Korean angelica fields in Eumseong, Korea (36.5°N, 127.4°E). Delta traps baited with tubes filled with test chemicals were hung from sticks at a height of 30 cm.

In addition, we evaluated the effect of addition of (*E*)-2-octenyl butyrate (E2OB) to the basic blends of hexyl butyrate (HB), (*E*)-2-hexenyl butyrate (E2HB) and (*E*)-4-oxo-2-hexenal (4-OHE) optimized for each of five mirid species during September 2014. Field trials for *A*. *lucorum*, *A*. *spinolae* and *T*. *apicalis* were conducted in vineyards in Suwon by using the basic blends of 100:20:20, 20:100:20 and 100:5:20 mixtures of HB, E2HB and 4-OHE, respectively. Field trials for *O*. *campestris* were carried out in Korean angelica fields in Eumseong and a 100:10:20 mixture of HB, E2HB and 4-OHE was used as the basic blend. To determine the effect of addition of E2OB to basic blend (100:20:20) for *S*. *rubrovittatus*, experiment was conducted in paddy fields in Icheon, Korea (37.1°N, 127.2°E).

All field experiments employed a randomized complete block design with three replicates of each treatment. Traps were placed about 10 m apart within a block, and blocks were separated by at least 100 m. Captured bugs were counted every 2–3 days, after which sticky inserts were replaced. *A*. *lucorum* and *A*. *spinolae* males caught were identified based on the structures at head part and tylus coloration patterns [19]. Moreover, Ram Keshari Duwal (Seoul National University, Korea) confirmed the identity of the specimens on the basis of the morphological investigation of the male genitalia [20]. Voucher specimens of *A*. *lucorum* and *A*. *spinolae* males were deposited in the entomological collection at National Institute of Horticultural and Herbal Science.

Trap catch data (*x*) were transformed to √(*x*+0.5) to normalize the variance and submitted to one-way analysis of variance (ANOVA) [21]. Treatments that failed to capture bugs were not included in the analyses to avoid violating assumptions of ANOVA. Means were ranked by Tukey’s HSD test at *α* = 0.05.

## Results

### Chemical Analyses

**Fig 1:**
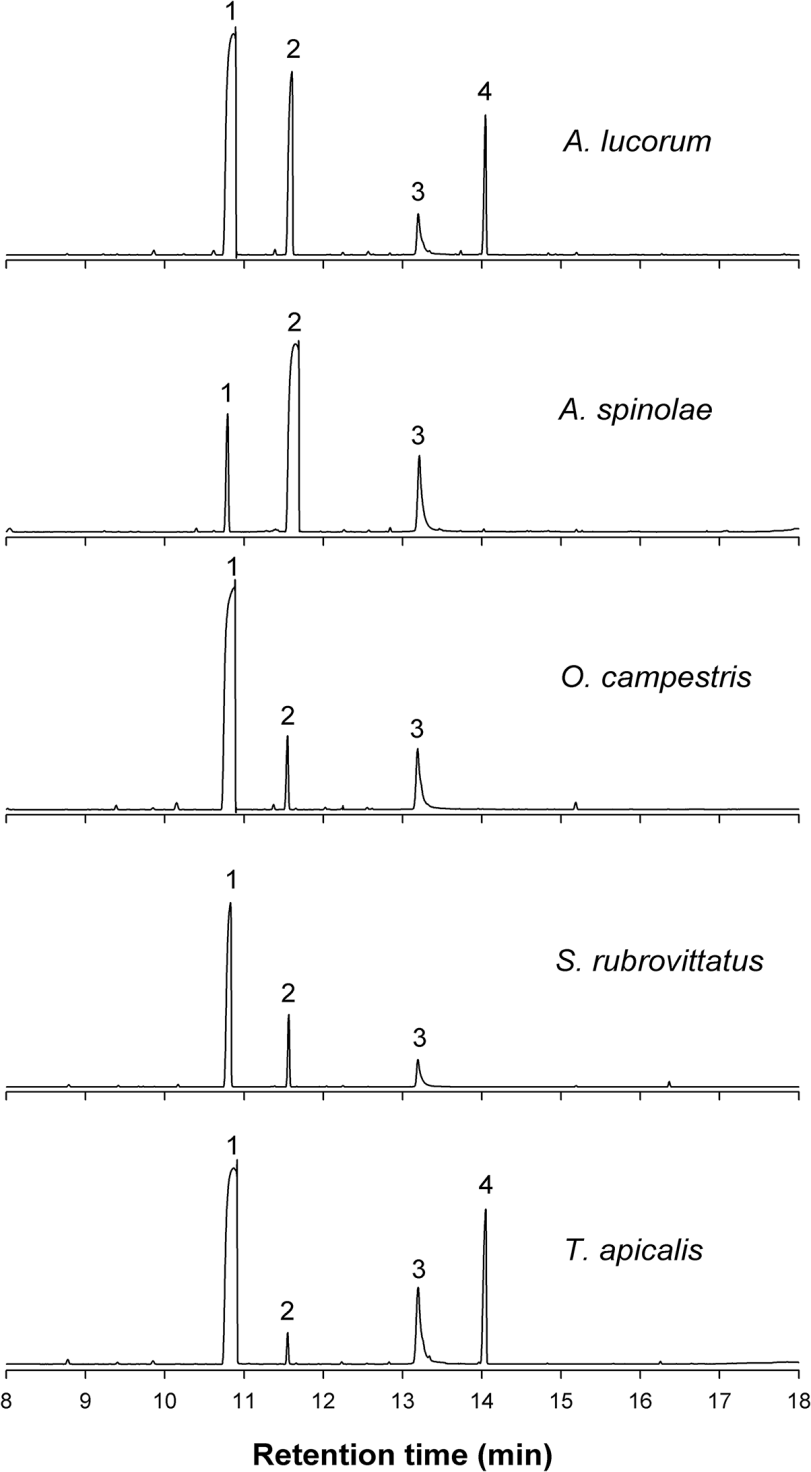
Total ion chromatograms of GC/MS analysis of metathoracic scent gland extracts from females of five mirid species on a DB-Waxetr column. Compound identities: 1 hexyl butyrate; 2 (*E*)-2-hexenyl butyrate; 3 (*E*)-4-oxo-2-hexenal; 4 (*E*)-2-octenyl butyrate.

GC/MS analyses showed that four major peaks (peaks 1, 2, 3 and 4) were detected in MSG extracts of *A*. *lucorum* and *T*. *apicalis* females, whereas three major peaks (peaks 1, 2 and 3) were found only in extracts of *A*. *spinolae*, *O*. *campestris* and *S*. *rubrovittatus* (Fig 1). The mass spectrum of peak 1 showed fragment ions *m/z* 129, 101, 89, 84, 71, 56 and 43 (base peak), and matched hexyl butyrate in a MS library search. Peak 1 had RIs indistinguishable from those of synthetic hexyl butyrate on both columns (DB-Waxetr: 1417, DB-23: 1441). The mass spectrum of peak 2 had a molecular ion at *m/z* 170, and fragment ions *m/z* 128, 100, 82, 71 (base peak), 55 and 43, indicating a hexenyl butyrate. The DMDS adduct of peak 2 showed a molecular ion *m/z* 264 (170 + MeSSMe) and diagnostic ions *m/z* 103 (C_3_H_7_CH = SCH_3_) and 161 (CH_3_S = CHCH_2_OCOC_3_H_7_), proving the addition of DMDS to a double bond at position 2 of the alcohol moiety [22]. The calculated RIs of peak 2 on the DB-Waxetr and DB-23 columns coincided with those of (*E*)-2-hexenyl butyrate (1483 and 1473, respectively) rather than (*Z*)-2-hexenyl butyrate (1467 and 1468, respectively).

The mass spectrum of peak 3 had fragment ions *m/z* 112 (M^+^), 83 (base peak) and 55, and matched (*E*)-4-oxo-2-hexenal in a MS library search. The RIs of the compound on both columns were identical to those of (*E*)-4-oxo-2-hexenal (DB-Waxetr: 1608, DB-23: 1621). Peak 4 showed diagnostic ions *m/z* 198 (M^+^), 155, 128, 110, 81, 71 (base peak), 54 and 43 suggestive of an octenyl butyrate. The mass spectrum of a DMDS adduct with M^+^ at *m/z* 292 (198 + MeSSMe) derived from the octenyl butyrate in MSG extract showed *m/z* 131 (C_5_H_11_CH = SCH_3_) and 161 (CH_3_S = CHCH_2_OCOC_3_H_7_), placing the double bond at position 2 of the alcohol moiety. The RIs of peak 4 on the DB-Waxetr and DB-23 columns were identical to those of (*E*)-2-octenyl butyrate (1677 and 1684, respectively), and markedly different from the RIs of (*Z*)-2-octenyl butyrate (1654 and 1647, respectively).

All species produced hexyl butyrate as a primary component, except for *A*. *spinolae*, which produced (*E*)-2-hexenyl butyrate as a main component (Table 1). Mean amounts of the primary component in extracts of *A*. *lucorum*, *O*. *campestris*, *S*. *rubrovittatus* and *T*. *apicalis* were 11.7, 13.2, 5.5 and 10.9 μg per female, respectively. In *A*. *spinolae* extracts, the amount of the main component, (*E*)-2-hexenyl butyrate, was estimated to be 11.3 μg per female.

**Table 1:** The relative ratios (mean±SD) of hexyl butyrate (HB), (*E*)-2-hexenyl butyrate (E2HB), (*E*)-4-oxo-2-hexenal (4-OHE) and (*E*)-2-octenyl butyrate (E2OB) found in metathoracic scent gland extracts from females of five mirid species (*N* = 10).

Species	Host plant	Relative ratio (%)			
		HB	E2HB	4-OHE	E2OB
*Apolygus lucorum*	Mugwort	100	23.6±10.2	12.3±5.2	16.8±6.5
*Apolygus spinolae*	Mugwort, Grape	20.6±10.4	100	13.4±5.4	-^a^
*Orthops campestris*	Angelica	100	9.7±2.4	16.6±5.0	-
*Stenotus rubrovittatus*	Rice	100	17.4±5.6	14.1±2.0	-
*Taylorilygus apicalis*	Mugwort	100	5.0±3.4	15.9±2.3	12.6±7.6

^a^ Not detected

### Field Trials

**Fig 2:**
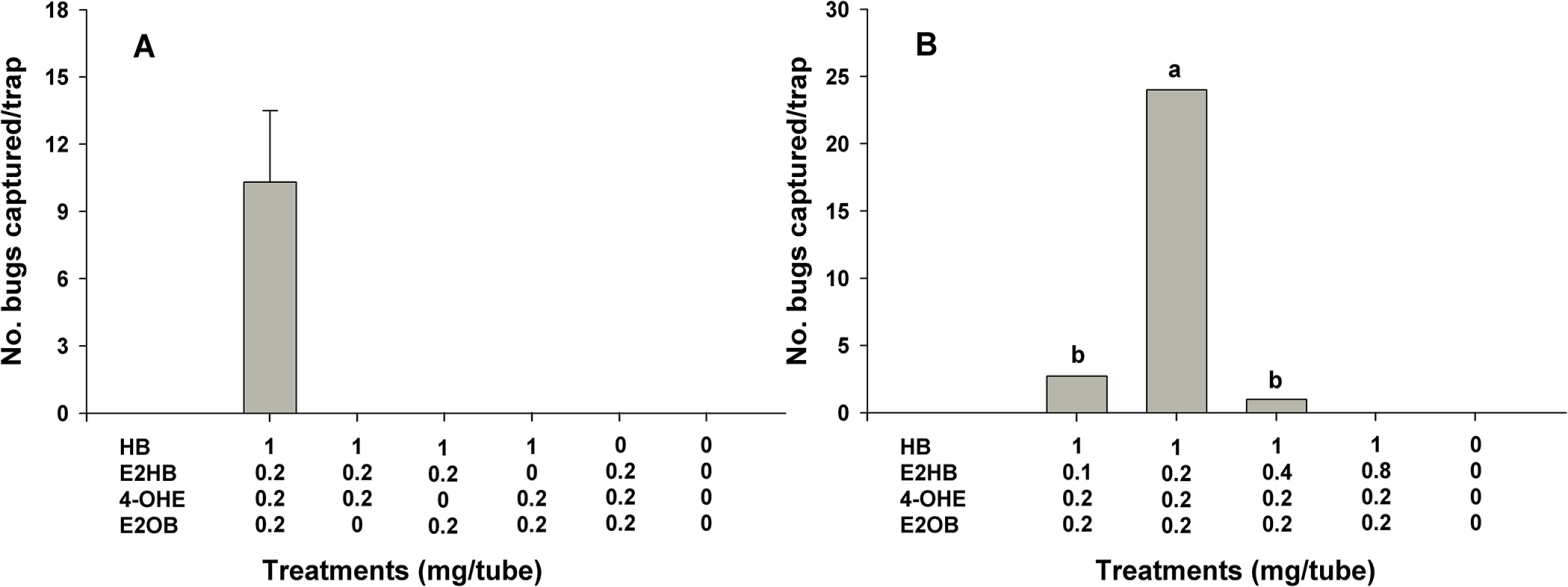
Mean catches of male *Apolygus lucorum* in traps baited with hexyl butyrate (HB), (*E*)-2-hexenyl butyrate (E2HB), (*E*)-4-oxo-2-hexenal (4-OHE) and (*E*)-2-octenyl butyrate (E2OB) in the quaternary blend and in the four possible tertiary blends (A; May 17–24, 2014) and in quaternary blends with varying amounts of E2HB (B; June 16–23, 2014) in vineyards in Suwon, Korea (*N* = 3). Bars with the same letter are not significantly different at *α* = 0.05 by Tukey’s HSD test. For A, mean + SD; and for B, *F* = 39.87; *df* = 2,4; *P*<0.001.

Preliminary field trials in 2013 that used single components and all possible binary blends of HB, E2HB, 4-OHE and E2OB in the ratios found in female extracts failed to attract *A*. *lucorum* males. Consequently, various ternary and quaternary blends of these compounds were tested in the two field trials in 2014. The quaternary blend mimicking the ratio found in a female extracts was attractive to male *A*. *lucorum*, but traps baited with the ternary blends missing any of the four components from the quaternary blend caught no males (Fig 2A). A subsequent field trial testing the effect of different ratios of E2HB revealed that the quaternary blend at a ratio of 100:20:20:20 caught significantly greater numbers of male bugs than any of the other lures tested (Fig 2B).

**Fig 3:**
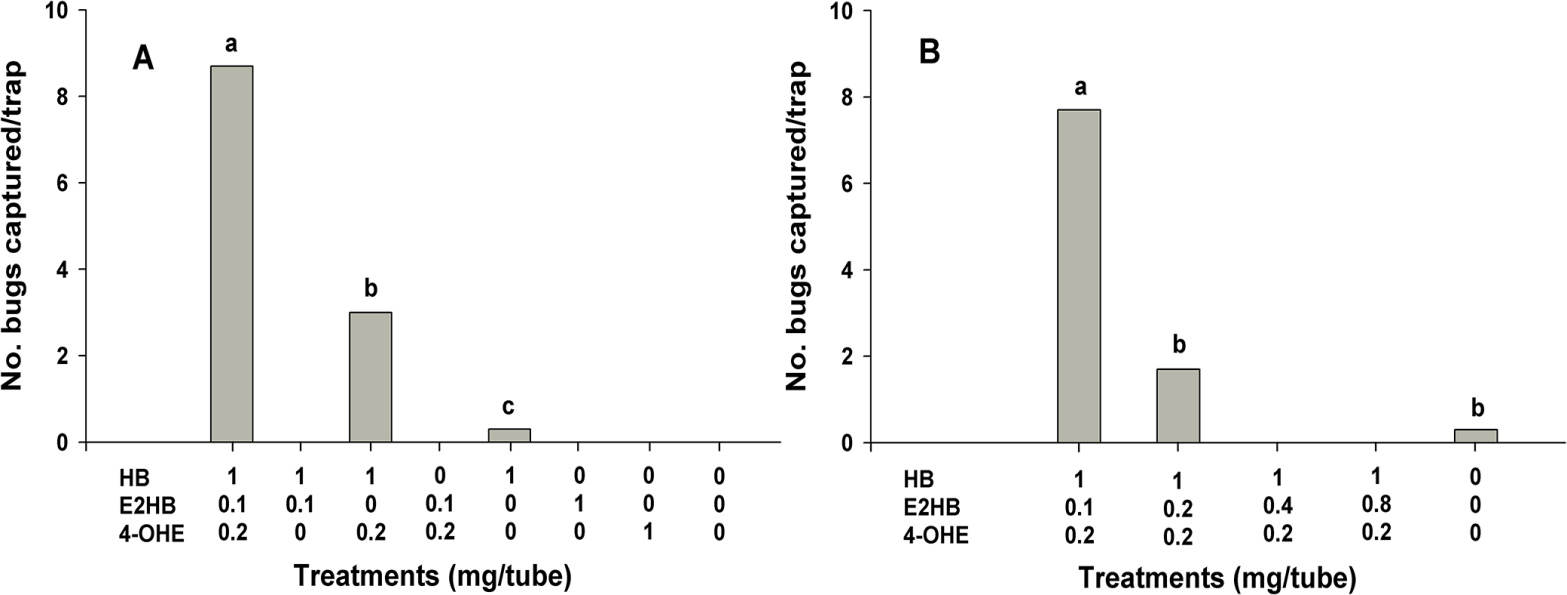
Mean catches of male *Orthops campestris* in traps baited with hexyl butyrate (HB), (*E*)-2-hexenyl butyrate (E2HB) and (*E*)-4-oxo-2-hexenal (4-OHE) singly, in the three possible binary blends or the tertiary blend (A; June 27-July 8, 2014) and in tertiary blends with varying amounts of E2HB (B; July 23–29, 2014) in angelica fields in Eumseong, Korea (*N* = 3). Bars with the same letter are not significantly different at *α* = 0.05 by Tukey’s HSD test. For A, *F* = 21.84; *df* = 2,4; *P*<0.01; and for B, *F* = 24.68; *df* = 2,4; *P*<0.01).

In the first field test for *O*. *campestris*, we found that the binary blend of HB and 4-OHE attracted a few males, but the ternary blend of HB, E2HB and 4-OHE was necessary for optimal attraction (Fig 3A). However, the second field test revealed that the addition of excessive amounts of E2HB to HB and 4-OHE mixtures significantly reduced attraction of *O*. *campestris* males (Fig 3B).

**Fig 4:**
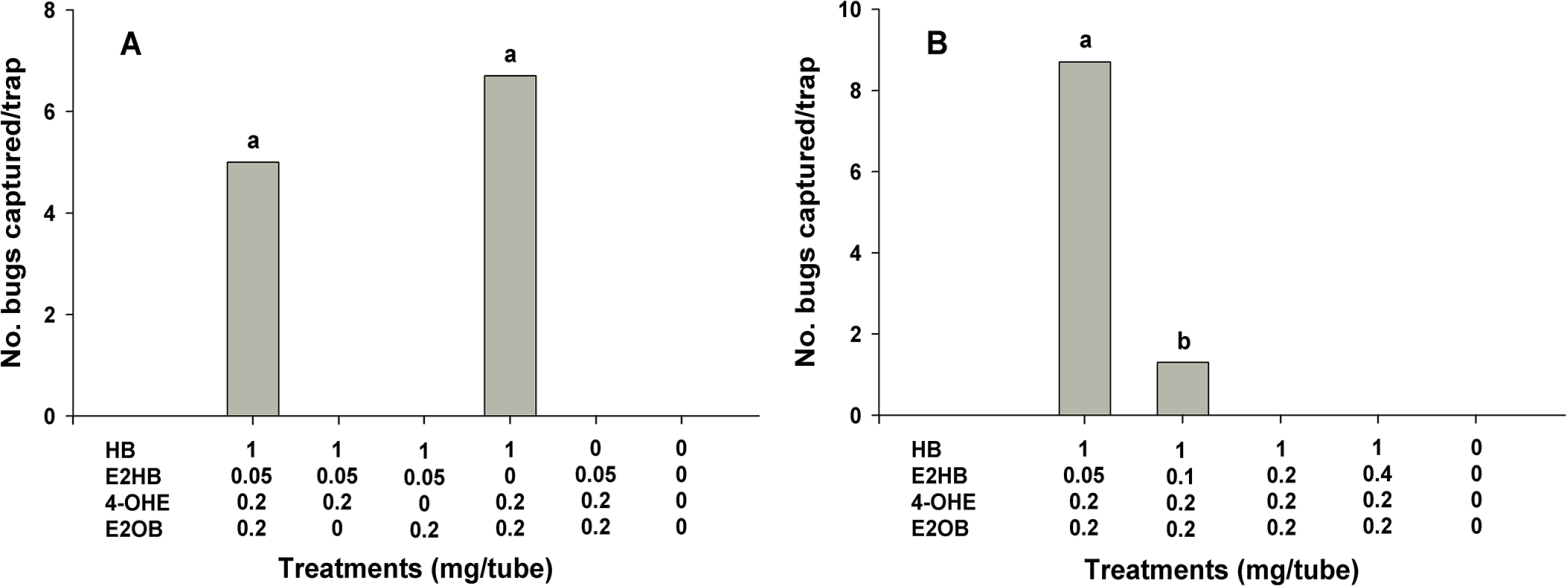
Mean catches of male *Taylorilygus apicalis* in traps baited with hexyl butyrate (HB), (*E*)-2-hexenyl butyrate (E2HB), (*E*)-4-oxo-2-hexenal (4-OHE) and (*E*)-2-octenyl butyrate (E2OB) in the quaternary blend and in the four possible tertiary blends (A; May 17–24, 2014) and in quaternary blends with varying amounts of E2HB (B; June 16–23, 2014) in vineyards in Suwon, Korea (*N* = 3). Bars with the same letter are not significantly different at *α* = 0.05 by Tukey’s HSD test. For A, *F* = 0.76; *df* = 1,2; *P* = 0.43; and for B, *F* = 78.00; *df* = 1,2; *P*<0.001.

As in *A*. *lucorum*, preliminary field tests for *T*. *apicalis* using single components and all possible binary blends of HB, E2HB, 4-OHE and E2OB failed to attract *T*. *apicalis* males. In subsequent experiments, we noticed that three components, HB, 4-OHE and E2OB, in the ratios found in female extracts are necessary for maximum attraction of males (Fig 4A). In contrast, adding E2HB at 10% or more of HB to the three-component attractive blend suppressed the attraction of male *T*. *apicalis* (Fig 4B).

**Fig 5:**
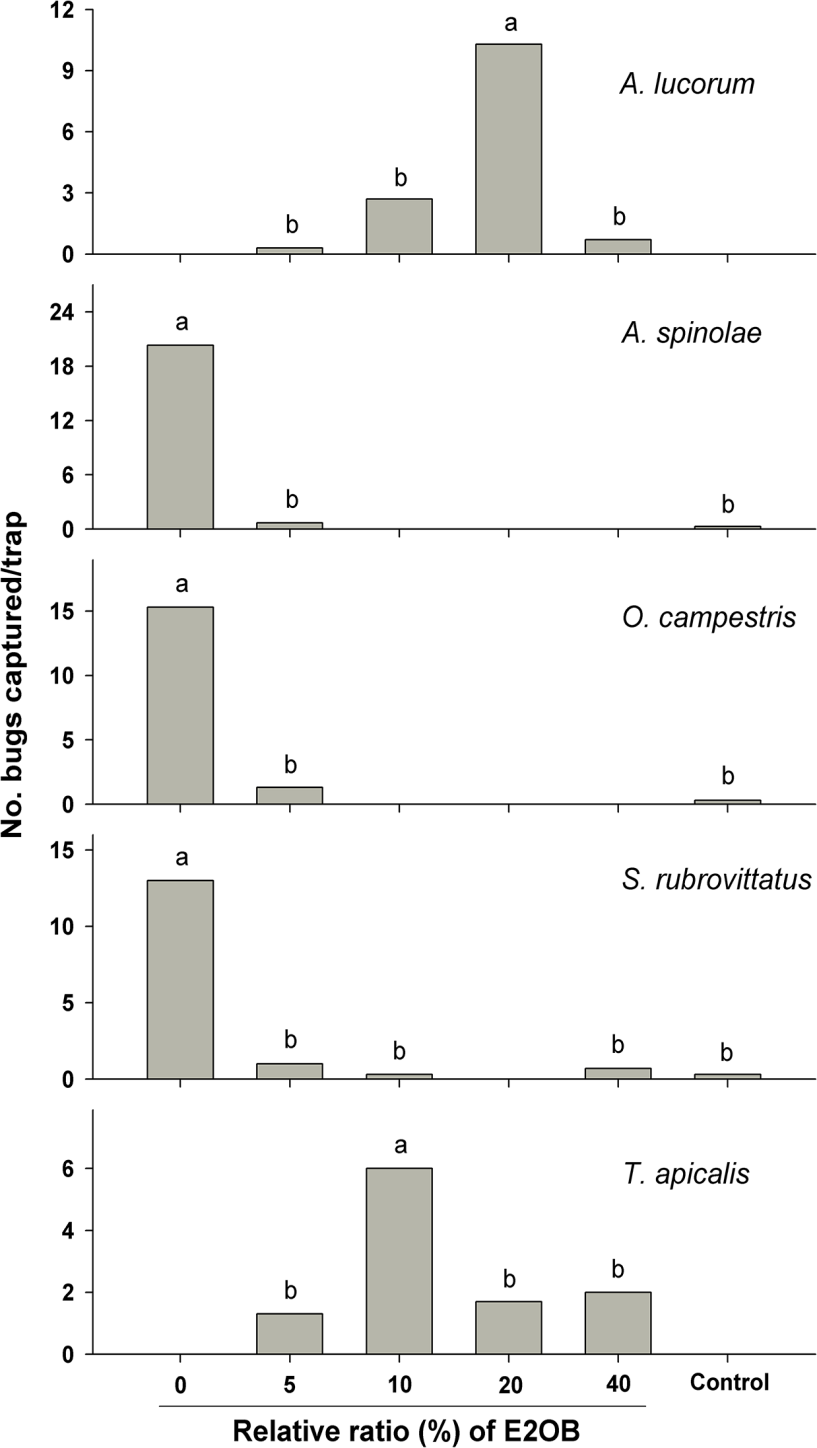
Effect of addition of E2OB at different doses to basic blends of HB, E2HB and 4-OHE on captures of male *Apolygus lucorum* (basic blend = 100:20:20, Suwon, September 11–19, 2014), *Apolygus spinolae* (basic blend = 20:100:20, Suwon, September 11–19, 2014), *Orthops campestris* (basic blend = 100:10:20, Eumseong, September 5–12, 2014), *Stenotus rubrovittatus* (basic blend = 100:20:20, Icheon, September 1–10, 2014) and *Taylorilygus apicalis* (basic blend = 100:5:20, Suwon, September 11–19, 2014) in Korea. Bars with the same letter are not significantly different at *α* = 0.05 by Tukey’s HSD test. For *A*. *lucorum*, *F* = 15.78; *df* = 3,6; *P*<0.001; for *A*. *spinolae*, *F* = 34.79; *df* = 2,4; *P*<0.01; for *O*. *campestris*, *F* = 44.10; *df* = 2,4; *P*<0.01; for *S*. *rubrovittatus*, *F* = 14.53; *df* = 4,8; *P*<0.001; and for *T*. *apicalis*, *F* = 10.23; *df* = 3,6; *P*<0.01. Control = blank trap.

The effects of addition of varying amounts of E2OB to the ternary blends of HB, E2HB and 4-OHE on trap catches varied significantly among mirid species (Fig 5). Blends missing E2OB were completely unattractive to *A*. *lucorum* and *T*. *apicalis* males, clearly indicating that E2OB is a critical component of the blends. *A*. *lucorum* males were significantly attracted to the quaternary blend containing 20% E2OB of HB, while male *T*. *apicalis* significantly preferred E2OB ratio of 10% of HB. On the other hand, addition of as little as 5% of E2OB to the ternary blend of HB, E2HB and 4-OHE inhibited attraction of *A*. *spinolae*, *O*. *campestris* and *S*. *rubrovittatus* males.

## Discussion

HB, E2HB and 4-OHE have been found in extracts of many mirid species, and have diverse functions such as defensive allomones, anti-sex pheromones, or sex pheromones [4]. These three compounds have been identified as components of the female sex pheromones of three Asian mirids, *S*. *rubrovittatus* [12], *A*. *spinolae* [13] and *Adelphocoris fasciaticollis* [23], three North American mirids, *Lygus hesperus*, *Lygus lineolaris* and *Lygus elisus* [24], and four European mirids, *Lygus pratensis*, *Lygus rugulipennis*, *Lygocoris pabulinus* and *Liocoris tripustulatus* [25]. *A*. *spinolae*, *L*. *lineolaris* and *L*. *elisus* use E2HB as their major component. In contrast, HB is the major component of the sex pheromone of the other seven species. These findings suggest that the relative composition of the two major components is a factor in the reproductive isolation of the sympatric mirid species [24].

In addition to HB, E2HB and 4-OHE, a small amount of E2OB was identified in the MSG extracts of *A*. *lucorum* and *T*. *apicalis* females and subtraction of this compound from the full four-component blends completely eliminated their attractiveness to male bugs. Therefore, the four-component pheromone blend identified in this study for *A*. *lucorum* and *T*. *apicalis* is the most complex blend described to date for the mirid species [4]. Although Millar et al. [26] reported that hexyl acetate and E2OB are pheromone components of another mirid species *Phytocoris relativus*, E2OB has never been identified from any mirid species which utilize HB, E2HB and 4-OHE as pheromone components. Consequently, our results may provide a broader insight into the evolution of pheromone communication in mirid species.

The combined results from this study and previous studies with *A*. *spinolae* and *S*. *rubrovittatus* suggest that species-specific blends of pheromone components are responsible for premating reproductive isolation between sympatric mirid species. For example, the addition of relatively large amounts of HB to the blend of E2HB and 4-OHE significantly decreased attraction of male *A*. *spinolae* [13]. Thus, *A*. *spinolae* males may not be attracted to female *O*. *campestris* and *S*. *rubrovittatus* that emit HB as their major pheromone component. In contrast, for *O*. *campestris* (this study, Fig 3B) and *S*. *rubrovittatus* [12], the addition of relatively large amounts of E2HB to the binary blend of HB and 4-OHE significantly decreased attraction of males. Therefore, males of these two mirids may not be attracted to *A*. *spinolae* females that release E2HB as its major pheromone component.

As noted above, E2HB was an essential component of the sex pheromone blend of *A*. *lucorum*. However, despite being present in gland extracts, it does not appear to be part of the attractive blend for *T*. *apicalis*. Moreover, it was antagonistic to *T*. *apicalis* at a level (ca. 20%) found in glands in *A*. *lucorum*, suggesting that E2HB plays an important role in reproductive isolation between *A*. *lucorum* and *T*. *apicalis* that utilize the same host-plant (mugwort).

Female *A*. *lucorum* and *T*. *apicalis* produced E2OB in addition to HB, E2HB and 4-OHE, and E2OB was essential for attraction of male *A*. *lucorum* and *T*. *apicalis*. Therefore, it could mean that *A*. *lucorum* and *T*. *apicalis* males would not be attracted to *A*. *spinolae*, *O*. *campestris* and *S*. *rubrovittatus* females because they do not emit the essential component, E2OB. On the other hand, *O*. *campestris* and *S*. *rubrovittatus* males would not be attracted to *A*. *lucorum* and *T*. *apicalis* females because the amount of E2OB they emit relative to HB acts as a behavioral antagonist.

The results show that *O*. *campestris* and *S*. *rubrovittatus* have similar pheromone systems comprised of HB, E2HB and 4-OHE. Yasuda et al. [18] reported that male *S*. *rubrovittatus* were less attracted by high doses of the ternary blend of HB, E2HB and 4-OHE in the field [12], but we found that attraction of *O*. *campestris* males to traps increased with an increase in loading of the ternary blend over the range from 0.01 to 10 mg (data not shown). More importantly, the quantity of the major compound, HB, in *O*. *campestris* gland extracts (13.2 μg/female) was significantly greater than that in *S*. *rubrovittatus* gland extracts (5.5 μg/female). These results indicate that pheromone concentration may be responsible in part for maintenance of premating reproductive isolation between the two species. However, the quantity of pheromone components in female gland extract may not be indicative as to the pheromone emission rate [27,28]. Further studies are necessary to determine the differences in the pheromone titers and ratios between female effluvia and gland extracts of mirid species, and to compare the attractiveness of synthetic pheromone lures with those of live virgin females. Furthermore, additional research is required to determine the importance of nonchemical factors such as diel periodicity of pheromonal communication in their reproductive isolation.

Extracts from Japanese females of *S*. *rubrovittatus* contain HB, E2HB and 4-OHE as sex pheromone components in a ratio of approximately 100:46:5 [12]. This ratio is quite different from that of the Korean population of *S*. *rubrovittatus* observed in our study. This divergence may be due to difference in the extraction time of sex pheromone components. Yasuda et al. [12] extracted from whole insect body for 5 min, whereas we extracted from MSG for 1 min. As described above, 4-OHE is known to be degraded rapidly in solvent in the presence of macerated insect material [16]. Hence, the ratio of 4-OHE in extracts from Japanese population was likely to have been relative low. In addition, such variation may be due to genetic drift in geographically isolated populations or to reproductive character displacement via communication interference between closely related sympatric species [6,29,30].

Zhang [14] reported that a Chinese population of *A*. *lucorum* utilizes E2HB and 4-OHE as components of its sex pheromone. The pheromone system of Chinese *A*. *lucorum* is markedly different from that observed in our study, whereas the Chinese pheromone blend is similar to that of *A*. *spinolae* from grapes in Korea [13]. Despite the morphological similarity of these two sibling species, *A*. *lucorum* can be distinguished from *A*. *spinolae* based on tylus coloration patterns of adult bugs [19] and the structures of the male genitalia [20]. Further investigation of the different geographical populations of *A*. *lucorum* is necessary to clarify the pheromone communication system of this species.

## References

[1] Cassis G, Schuh RT. Systematics, biodiversity, biogeography, and host associations of the Miridae (Insecta: Hemiptera: Heteroptera: Cimicomorpha). Annu Rev Entomol. 2012;57: 377–404. 10.1146/annurev-ento-121510-133533 22149267

[2] Scales AL. Female tarnished plant bugs attract males. J Econ Entomol. 1968;61: 1466–1467.

[3] McBrien HL, Millar JG. Phytophagous bugs In: Hardie J, Minks K, editors. Pheromones of non-lepidopteran insects associated with agricultural plants. CABI Publishing, New York; 1999 pp. 277–304.

[4] El-Sayed AM. The Pherobase: database of insect pheromones and semiochemicals; 2015. Available: http://www.pherobase.com/database/family/family-Miridae.php.

[5] Roelofs WL, Brown RL. Pheromones and evolutionary relationships of Tortricidae. Annu Rev Ecol Evol Syst. 1982;13: 395–422.

[6] Löfstedt C, Herrebout WM, Menken SBJ. Sex pheromones and their potential role in the evolution of reproductive isolation in small ermine moths (Yponomeutidae). Chemoecology. 1991;2: 20–28.

[7] Yang CY, Han KS, Boo KS. Sex pheromones and reproductive isolation of three species in genus *Adoxophyes* . J Chem Ecol. 2009;35: 342–348. 10.1007/s10886-009-9602-z 19221842

[8] Hayashi H, Nakazawa K. Studies on the bionomics and control of the sorghum plant bug, *Stenotus rubrovittatus* Matsumura (Hemiptera: Miridae) 1. Habitat and seasonal prevalence in Hiroshima Prefecture. Bull Hiroshima Pref Agric Exp Stn. 1988;51: 45–53.

[9] Watanabe K. Damages and control of *Lygocoris* (*Apolygus*) *lucorum* (Meyer-Dür) (Heteroptera: Miridae) on cherry. Plant Prot. 1999;43: 56–59.

[10] Lee S, Lee GS, Goh HG. Mirid bugs (Heteroptera: Miridae) on grapevine: their damages and host plants. Korean J Appl Entomol. 2002;41: 33–41.

[11] Lu Y, Wu K, Wyckhuys KAG, Guo Y. Overwintering hosts of *Apolygus lucorum* (Hemiptera: Miridae) in northern China. Crop Prot. 2010;29: 1026–1033.

[12] Yasuda T, Shigehisa S, Yuasa K, Okutani-Akamatsu Y, Teramoto N, Watanabe T, et al Sex attractant pheromone of the sorghum plant bug *Stenotus rubrovittatus* (Matsumura) (Heteroptera: Miridae). Appl Entomol Zool. 2008;43: 219–226.

[13] Yang CY, Kim J, Ahn S, Kim D, Cho MR. Identification of the female-produced sex pheromone of the plant bug *Apolygus spinolae* . J Chem Ecol. 2014;40: 244–249. 10.1007/s10886-014-0407-3 24647725

[14] Zhang T. The study of the extraction, identification and application of sex pheromone produced by *Apolygus lucorum* Doctoral Dissertation, Chinese Academy of Agricultural Science. 2012. Available: http://www.globethesis.com/?t=1103330335479304.

[15] Moreira JA, Millar JG. Short and simple syntheses of 4-oxo-(*E*)-2-hexenal and homologs: pheromone components and defensive compounds of Hemiptera. J Chem Ecol. 2005;31: 965–968. 16124263 10.1007/s10886-004-1978-1

[16] Byers JA. Production and predator-induced release of volatile chemicals by the plant bug *Lygus hesperus* . J Chem Ecol. 2006;32: 2205–2218. 17001534 10.1007/s10886-006-9140-x

[17] Buser H, Arn H, Guerin P, Rauscher S. Determination of double bond position in mono-unsaturated acetates by mass spectrometry of dimethyldisulfide. Anal Chem. 1983;55: 818–822.

[18] Yasuda T, Mochizuki F, Yasuda M, Takeda A, Higuchi H, Watanabe T, et al Performance of polyethylene tubes as pheromone lures for the sorghum plant bug, *Stenotus rubrovittatus* (Hemiptera: Heteroptera: Miridae). Appl Entomol Zool. 2013;48: 325–330.

[19] Jung S, Duwal RK, Lee S. COI barcoding of true bugs (Insecta, Heteroptera). Mol Ecol Resour. 2011;11: 266–270. 10.1111/j.1755-0998.2010.02945.x 21429132

[20] Yasunaga T. A revision of the plant bug, genus *Lygocoris* Reuter from Japan, Part Ⅳ. (Heteroptera, Miridae, *Lygus*-complex). Jpn J Entomol. 1992;60: 10–25.

[21] SAS Institute. Statview. SAS Institute Inc., Cary; 2010.

[22] Kakizaki M, Sugie H. Identification of female sex pheromone of the rice leaf bug, *Trigonotylus caelestialium* . J Chem Ecol. 2001;27: 2447–2458. 11789951 10.1023/a:1013623414324

[23] Zhang T, Mei X, Zhang L, Wu K, Ning J. Identification of female sex pheromone of a plant bug, *Adelphocoris fasciaticollis* Reuter (Hemiptera: Miridae). J Appl Entomol. 2015;139: 87–93.

[24] Byers JA, Fefer D, Levi-Zada A. Sex pheromone component ratios and mating isolation among three *Lygus* plant bug species of North America. Naturwissenschaften. 2013;100: 1115–1123. 24233237 10.1007/s00114-013-1113-7

[25] Fountain M, Jåstad G, Hall D, Douglas P, Farman D, Cross J. Further studies on sex pheromones of female *Lygus* and related bugs: development of effective lures and investigation of species-specificity. J Chem Ecol. 2014;40: 71–83. 10.1007/s10886-013-0375-z 24390623

[26] Millar JG, Rice RE, Wang Q. Sex pheromone of the mirid bug *Phytocoris relativus* . J Chem Ecol. 1997;23: 1743–1754.

[27] Evenden ML, Gries R. Sex pheromone of the large aspen tortrix, *Choristoneura conflictana* (Lepidoptera: Tortricidae). Chemoecology. 2006;16: 115–122.

[28] Yang CY, Paik CH, Lee GH, Park JY. Female sex pheromone of the gelechiid moth *Scrobipalpa salinella* (Zeller). J Chem Ecol. 2011;37: 1216–1221. 10.1007/s10886-011-0026-1 22037791

[29] McElfresh JS, Millar JG. Geographic variation in sex pheromone blend of *Hemileuca electra* from southern California. J Chem Ecol. 1999;25: 2505–2525.

[30] Gries G, Schaefer PW, Gries R, Liška J, Gotoh T. Reproductive character displacement in *Lymantria monacha* from northern Japan? J Chem Ecol. 2001;27: 1163–1176. 11504021 10.1023/a:1010316029165

